# Nephrolepis exaltata Herbal Mask Increases Nasal IgA Levels and Pulmonary Function in Textile Factory Workers

**DOI:** 10.1155/2019/5687135

**Published:** 2019-12-09

**Authors:** Awal Prasetyo, Arindra Adi Rahardja, Dhiva Tsuroya Azzahro, Ika Pawitra Miranti, Indah Saraswati, Fathur Nur Kholis

**Affiliations:** ^1^Department of Biomedical Science, Faculty of Medicine, Diponegoro University, Indonesia; ^2^Undergraduate Student of Medical Faculty, Diponegoro University, Semarang, Indonesia; ^3^Department of Anatomic Pathology, Faculty of Medicine, Diponegoro University-Kariadi Hospital Semarang, Indonesia; ^4^Department of Medical Chemistry, Faculty of Medicine, Diponegoro University Semarang, Indonesia; ^5^Pulmonology Division of Internal Department Medical Faculty, Diponegoro University-Kariadi, Hospital Semarang, Indonesia

## Abstract

**Background:**

Chronic occupational exposure in textile workers lowers the pulmonary function and levels of sinonasal IgA. A *Nephrolepis exaltata* herbal mask can protect the respiratory tract. This study aims to understand the effect of this herbal mask on the IgA levels and pulmonary function in textile workers. Thirty employees were selected for this study.

**Methods:**

The pre- and post-test randomized experimental control trials were conducted in a garment industry of Bawen, Semarang, Indonesia. The subjects that qualified to participate (*n* = 30) fulfilled the inclusion criteria i.e., 20–35 years old, healthy, and willing to be a research subject; and exclusion criteria i.e., having history of alcohol consumption, smoking, history of liver disease, autoimmune disease, cancer, pulmonary and heart disease and/or being pregnant. The subjects were then divided randomly into control group (*n* = 15), who used regular mask that was rewashed and changed every month for eight weeks, and treatment group (*n* = 15), who used *Nephrolepis exaltata* mask that was changed every two days for eight weeks. Pulmonary function tests were carried out using MIR Spirolab III before and after the experiment. IgA levels were measured by nasal wash method using ELISA.

**Results:**

IgA levels of the treatment group before and after usage of mask were significantly different (*p*<0.001) compared to the control group. There were significant difference in FVC of the control group, but no significant difference was observed for FEV1 (*p* = 0.507) and PEF (*p* = 0.001). In the treatment group, all three parameters showed significant differences [FVC (*p* = 0.038), FEV1 (*p* = 0.004), and PEF (*p* = 0.001)]. The means of ΔFVC, ΔFEV1, and ΔPEF were significantly (*p*<0.05) higher in the treatment group with OR = 5.1 for higher IgA levels.

**Conclusions:**

The herbal mask is better in increasing IgA and improving the pulmonary function compared to the regular mask.

## 1. Introduction

A large amount of chemical material are used in the process of textile manufacture. The chemicals in the working environment, particularly volatile organic compounds (VOC), are constantly inhaled by the workers. The exposure of respiratory tract to these irritant compounds can cause hyperreactivity of mucous membrane resulting in inflammation of the mucosa, obstruction, restriction, as well as both of them to manifest decreased pulmonary function. Besides VOC, formaldehyde is generally found in the work environment in gaseous form. Its particle size is undetermined and but its density is 1.067 relative to air (air = 1) with a molecular mass of 30.03 g/mol [18]. In some case reports and clinical studies, mild to medium stages of irritation in the eyes, nose, and throat were observed in volunteers who were exposed to 0.25–3 ppm formaldehyde for a short period of time. Formaldehyde is an irritant of the respiratory tract, and some cases demonstrated that occurrence of bronchial asthma can be correlated to inhalation of formaldehyde [1]. The first component of the immune system that works on mucosal region is immunoglobulin A (IgA) which is the predominant immunoglobulin in mucosa, and also the second most substantial immunoglobulin in the serum. After formation of immunoglobulin A dimer, it is bound by secretory components (SC) such as saliva, and mucus to form Secretory IgA (SigA). This SigA inhibits enzymes and slows down the colonization of bacteria on the mucosal surfaces [2].

While VOC, a chemical material usually in gaseous form can irritate the respiratory tract, the same symptoms may occur due to exposure to organic materials derived from food processing, such as seafood, especially crustaceans (arthropods with chitin exoskeleton, for example, crab, lobster, shrimp, etc.). The allergen can come into contact with the body through ingestion, direct skin contact, or inhalation of the allergen, thereby causing allergic reaction. Although both chemical or organic materials may cause similar respiratory symptoms, allergic reaction from organic material usually occurs after sensitization phase and development of specific immunoglobulin E (IgE) antibodies. In sensitization phase, subjects may come into contact with the allergen for a long period of time without developing allergic symptoms, this makes the subjects oblivious to the allergen source. The subjects will start showing allergic symptoms in re-exposure phase, the symptoms such as stuffy nose, sneezing, rhinorrhea, itching, and watery red eyes are commonly observed. Meanwhile, chemical materials may cause similar symptoms, but with milder or non-existent sneezing and nose itching. Blood test can be used to detect IgE increase in allergic symptoms [16, 17].

Decreased pulmonary function may reduce the quality of workers' life and may also affect their productivity at work [3, 4]. Therefore, efforts to protect respiratory organs from chemical exposure at work environment are highly important. Mouth and nose must be covered by mask to filter entering particles of dust/vapor at work place. Currently used masks, cloth masks and activated carbon filter masks, are not effective for the workers of textile factories who are exposed to dust/vapor. Regular cloth mask cannot prevent the entry of chemicals in gas/vapor form into respiratory tract. Activated carbon mask has better filtering/reduction ability; however, it is not frequently used as it is not affordable. This difference in price can be attributed to difference in the quality of filters. The filters used in modern surgical masks and respirators are considered “fibrous” in nature; constructed from flat, nonwoven mats of fine fibers. Fiber diameter, porosity (the ratio of open space to fibers) and filter thickness all play a role in how well a filter collects particles. Cloth mask or surgical mask have varying pore sizes, with the average pore size ranging from 16.9–51.0 microns [5, 19].

Various attempts are constantly donein order to find affordable protective equipments which are highly effective in protecting respiratory system from the VOC exposure. One of the alternatives that can be used as herbal mask, is made from *Nephrolepis exaltata* [6]. Previous study by NASA (National Aeronautics and Space Administration) has shown that *Nephrolepis exaltata* can absorb formaldehyde, xylene, trichloroethylene, and carbon monoxide from the air [7]. Moreover, *Nephrolepis exaltata* has pore opening ranging from 3.5–10 microns and essential oils that possess antioxidant and anti-inflammatory properties [6, 20]. According to our previous research, *Nephrolepis exaltata* herbal mask showed better protection capabilities in the Sprague Dawley rats exposed to BTEX vapor than the masks with activated carbon filters. The number of goblet cells after BTEX vapor exposure in the group that employed *Nephrolepis exaltata* herbal mask was less than that in the group that employed activated carbon filter masks. This potential can be used as a new idea in the field of medicine to facilitate the manufacture of herbal masks for protecting respiratory organs from exposure to chemicals in their work environment. The benefits of *Nephrolepis exaltata* herbal mask has not been studied in detail. Hence, further research needs to be carried out to prove that this herbal mask can be applied as a protective equipment for human respiratory system to prevent exposure to dangerous chemicals, especially VOC at work environment and to protect pulmonary function [6].

In Indonesia, *Nephrolepis exaltata* is considered as an invasive plant, as it can proliferate rapidly and easily grow in cool and humid conditions. *Nephrolepis exaltata* not only can be found in the woods and swamp regions, but also can be found inside houses and gardens. This plant is easy to maintain since it does not require excessive fertilizers. In addition, it can tolerate dryness, grow better in cool, and slightly moist places, even when indirectly exposed to light. In few studies, *Nephrolepsis exaltata* is included in plants that have high efficiency to absorb air pollutants including formaldehyde [8–11].

This research was aimed to understand the effects of *Nephrolepis exaltata* herbal protection mask on IgA level and pulmonary function in textile factory workers. The results of this study are expected to provide information about the benefits of *Nephrolepis exaltata* herbal mask, which can be used to improve occupational health, and as a foundation for further research.

## 2. Materials and Methods

This was an experimental study with pre- and post-test randomized control trials. Samples were the employees of a garment industry in Bawen, Semarang, Indonesia who were exposed to occupational chemicals in the dyeing process section. Based on inclusion criteria i.e., 20–35 years old, healthy, and willing to be a research subject; and exclusion criteria i.e., having history of alcohol consumption, smoking, liver disease, autoimmune disease, cancer, pulmonary and heart disease and/or being pregnant; 30 employees were selected. They were divided into two groups, namely, group with *Nephrolepis exaltata* herbal mask and control group with regular cloth mask. The treatment group who used *Nephrolepis exaltata* mask was changed every two days for eight weeks, whereas the control group who used regular cloth mask was rewashed and changed every month for eight weeks. The sampling was carried out by nasal wash methodology after eight weeks of mask usage.

### 2.1. IgA Levels Assay

Sampling was performed before the treatment was given to the subject samples. The sampling used nasal wash methodology as described by Naclerio et al. [12]. Firstly, the subject was instructed to sit with his head extended at 45°. Then he was instructed to take a deep breath and hold his breath. Afterwards, a syringe containing 5 ml of isotonic solution which had been warmed to 37°C was put into one of his nostrils while another nostril was closed. The subject was asked to hold his position for a few seconds, and then to bow and gently remove the liquid into a container. This procedure was applied to another nostril as well.

After the above procedure, samples were assessed using ELISA (Enzyme-Linked Immunosorbent Assay) methodology with Human IgA (Immunoglobulin A) ELISA Kit from Elabscience® (catalog number E-EL-H1355). The samples taken pre- and post-test were preserved in an ice box and shifted to a refrigerator to be deposited for 24 h. The samples were then centrifuged and 1 ml of the supernatant was transferred to a cuvette. The samples were frozen and subsequently read only after calibration and tool optimization [13].

The difference in IgA levels of the control group before and after using mask were examined using Wilcoxon test since the data was not distributed normally. The difference in IgA levels of the treatment group was examined using paired *t*-test since the data was distributed normally.

### 2.2. Spirometer Measurement

Pulmonary function parameters measured in this study were Forced Vital Capacity (FVC), Forced Expiratory Volume in One Second (FEV_1_), and Peak Expiratory Flow (PEF). Measurement of pulmonary function parameters was done using Medical International Research Spirolab III portable spirometer before and after using mask. Data distribution was analyzed using data normality test called Shapiro–Wilk test. The mean differences between FVC, FEV_1_, and PEF values before and after treatment in both groups were analyzed using paired *t*-test since all data showed normal distribution. The difference in pulmonary function parameters in both groups were analyzed using the Mann–Whitney test and the nonparametric different test since the data did not show normal distribution.

Ethical clearance was obtained from Commission on Health Research Ethics, Faculty of Medicine, Diponegoro University and Dr. Kariadi Hospital, Semarang, Indonesia (No. 461/EC/FK-RSDK/VII/2017).

## 3. Results

### 3.1. Subject Characteristics

Analysis of research subjects showed that variables such as age, sex, BMI, length of work, history of respiratory disease, allergy and smoking were not statistically different (*p*>0.05) between control and treatment groups. Therefore, the subjects were eligible for different tests conducted (IgA and pulmonary function parameters), However, the history of smoking was almost significant for the two groups (*p* = 0.06) (Table 1).

### 3.2. IgA Level

**Figure 1:**
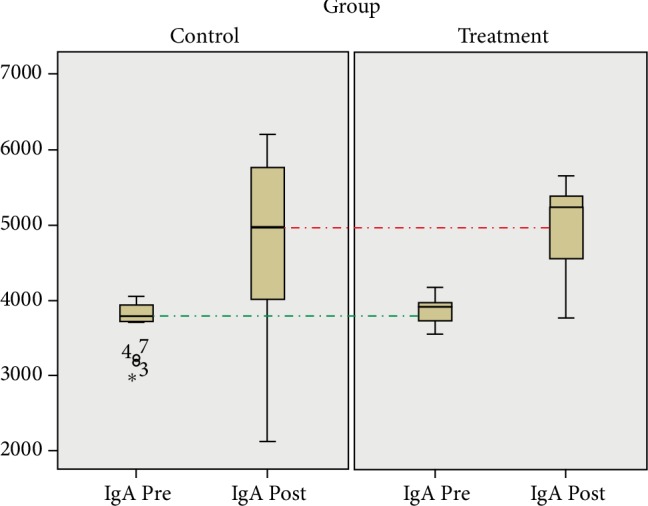
Boxplot IgA level before and after treatment.

The mean IgA level before and after using regular cloth masks showed increase from 3691.5 ± 325.02 to 4656.9 ± 1345.66. The Wilcoxon test showed that the increase in IgA level was statistically significant (*p*<0.05). The increased mean IgA level was also observed after using *Nephrolepis exaltata* herbal mask, from 3838.4 ± 172.3 to 4935.88 ± 608.01. The paired *t*-test showed that the increase in IgA level was significantly different between the two groups. The delta of the control group was lower than that of the treatment group with the odds ratio to have lower IgA levels five-fold higher for the control group (Table 2 and Figure 1).

**Figure 2:**
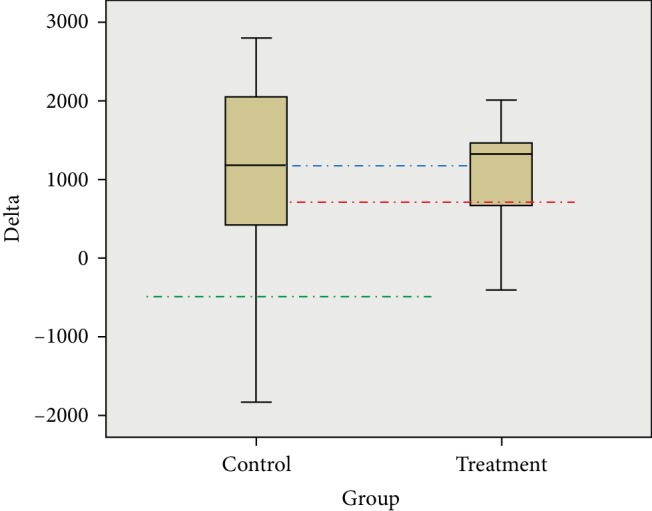
Mean of delta score of IgA level between two groups.

IgA level was found to be significantly increased after treatment (*p* < 0.001). The mean IgA level in control and treatment groups increased to 4656.9 ± 1345.67 and 4935.9 ± 608.01, respectively. The result showed that there was no significant difference in IgA level between treatment and control groups (*p* = 0.745). However, the mean difference for the control group was 965.3 ± 1411.37, with minimum and maximum differences −1831.6 and 2800.1, respectively. The mean difference for the treatment group was 1097.5 ± 656.16, with minimum and maximum differences −410.9 and 2011.8, respectively (Figure 2).

### 3.3. Pulmonary Function Parameters

There was a significant decrease of FVC score in the control group (paired *t*-test, *p* = 0.033). In contrast, FVC score in the treatment group showed a significant increase (paired *t*-test, *p* = 0.038). Mann–Whitney test was performed to test the significance of difference in FVC between two unpaired groups, namely control and treatment groups. A significant difference (*p* = 0.006) was observed in ΔFVC before and after treatment between control and treatment groups (Table 3).

There was a decrease in FEV_1_ score of the control group (paired *t*-test, *p* = 0.507). In contrast, FEV_1_ score in the treatment group showed a significant increase (paired *t*-test *p* = 0.004). Mann–Whitney test showed significant difference (*p* = 0.009) in ΔFEV_1_ before and after treatment between control and treatment groups (Table 4).

A decrease in PEF score of the control group (paired *t*-test, *p* = 0.231) was also observed. In contrast, PEF score of the treatment group showed a significant increase (paired *t*-test, *p* = 0.001). Mann–Whitney test showed significant difference (*p* = 0.001) in ΔPEF before and after treatment between control and treatment groups (Table 5).

## 4. Discussion

The present study found that wearing a *Nephrolepis exaltata* herbal mask for eight weeks can normalize IgA level of sinonasal mucosa. Previous study by Kwang et al. and Wolverton et al. showed that *Nephrolepis exaltata* can absorb and neutralize VOC. Consistent with this finding, our previous research showed that this plant significantly reduces the number of sinonasal-goblet cells in Sprague Dawley rats which were exposed to VOC [6].

In this study, fifteen control subjects used cloth masks which failed to provide sufficient protection from small pollutants. Usually, pollutants with small diameter (≤2.5 mm) are inhaled until they reach the lungs. Jeremi et al. showed that cloth mask was only able to filter 28% pollutants in highly polluted areas, such as highways in China [14].

*Nephrolepis exaltata* is cheap, easy to obtain, easy to maintain and easy to proliferate plant. Thus, it can be independently cultivated. This plant contains substances that are useful to neutralize air pollutants such as formaldehyde, benzene, toluene, xylene, and many others VOC. Previous study by Kwang et al. (2010) showed that the filtration property of *Nephrolepis exaltata* is very effective in neutralizing VOC. Our previous study on Sprague Dawley rats also demonstrates the potency of *Nephrolepis exaltata* in filtering BTX compound, which belongs to VOC [6, 10, 15].

IgA levels are known to increase with acute infection and inflammation. The exposure of textile factory workers to chemical materials, such as allergens or irritants, especially in the dyeing area, may increase acute and chronic inflammatory response. IgA level of the subject which was low in pre-test phase may increase by chronic exposure towards post-test phase. The subject was found to develop flu during post-test phase. The flu developed acutely for a few days before the post-test phase. Flu or acute rhinitis can trigger acute inflammation, thereby increasing IgA levels in the infected target. Previous study by Deo et al. (2004) has shown that acute infection can lead to IgA elevation. The flu observed in the subject at the time of post-test may affect the results of this study.

Chronic exposure to chemicals causes respiratory edema and increased mucous secretion resulting in narrowing of the respiratory tract. The pulmonary parenchyma also gets inflamed, resulting in reduced lung capacity and elasticity. Inflammation of respiratory tract and pulmonary parenchyma may cause decreased pulmonary function, which can be measured by a spirometer. The dyeing sector work environment typically has high humidity, where the effects of exposure to hazardous chemicals can be further increased. The effect of such exposure on pulmonary function is reversible and returns to normal after four weeks without exposure.

The decreased pulmonary function in the control group was suspected due to constant inhalation of chemicals in the work environment. Masks used by the control group were regular cloth masks, which were only able to prevent the entry of large particles, while chemical pollutants in gaseous form were able to penetrate and enter the respiratory tract of the workers.

The results obtained are in accordance with the research hypothesis stating that there is no increase in the pulmonary function after using regular cloth mask. Therefore, the above hypothesis can be accepted. The treatment group used herbal mask that contains essential oils and amines. It is well-known that essential oils act as antioxidants and anti-inflammatory agents that can be used in fighting against oxidative stress and inflammation caused by inhalation of chemicals. This is consistent with the findings by Kfoury et al. (2016) who reported the inhibition of inflammatory process in pulmonary and hepatic cells exposed to chemical pollutants after addition of essential oils. Amines in *Nephrolepis exaltata* can react with formaldehyde in work environment to form imines and water. As a result, formaldehyde which can potentially damage the structure and function of the cells cannot enter the respiratory tract. In a study in 1989, National Aeronautics and Space Administration (NASA) stated that besides formaldehyde, *Nephrolepis exaltata* can also absorb other chemical pollutants such as xylene, trichloroethylene, and carbon monoxide from the air. The exact mechanisms that play role in this process are not yet known. The properties of *Nephrolepis exaltata* are suspected to be the cause of improvement in lung function of the treatment group. Chemicals in the work environment were prevented from entering the respiratory tract by the *Nephrolepis exaltata* herbal mask used by the treatment group. Exposure of the respiratory organs to chemicals was reduced for eight weeks of treatment so that the decrease in pulmonary function that previously occurred could be restored.

The role of *Nephrolepis exaltata* mask in protecting respiratory system was also supported by the findings of Prasetyo et al. Their study was performed on Sprague Dawleyrats which were exposed to BTEX. The results showed that *Nephrolepis exaltata* herbal mask was better than activated carbon filtered mask in protecting respiratory organs of Sprague Dawley rats exposed to BTEX vapor. The number of goblet cells in group with *Nephrolepis exaltata* herbal mask was less than that in group with activated carbon filtered mask after being exposed to BTEX vapor [6].

The results obtained are in accordance with the research hypothesis stating that there is an improvement in pulmonary function after using *Nephrolepis exaltata* herbal mask. Therefore, the above hypothesis can be accepted. Opposite effects were observed with both types of mask used in this research. The use of regular cloth mask led to decreased pulmonary function, while the use of *Nephrolepis exaltata* herbal mask led to improved pulmonary function.

There were no side effects of using *Nephrolepis exaltata* herbal mask such as allergies. Adherence to usage and side effects of mask usage were supervised by the researchers by directly interviewing some of the research subjects. These parameters were also supervised by dyeing supervisors every two weeks.

The limitation of this study was that adherence to the use of personal protective equipment (masks) was less controllable in work environments. The data collection time of this study could not be equated to all samples. The differences in data collection time may be affected by physical and mental conditions of the samples, which may have significant impact on this study. This research was done on a limited population, i.e., only on the dyeing unit of one textile factory. In a larger population of textile industry workers with more samples, the result of the study may be differ. This can be attributed to differences in the conditions of the work environment and the adherence to usage of personal protective equipments in dyeing units.

## 5. Conclusion and Suggestions

Using a herbal mask may decrease IgA levels in the acutely exposed subject. This research also indicates that enhancement in IgA levels of subjects with chronic exposure results in restoration of IgA levels. *Nephrolepis exaltata* herbal mask users showed five-fold increase in IgA level compared to those who used regular cloth mask. Pulmonary function was found to be impaired after using regular cloth mask. The decrease in FVC score was statistically significant, whereas the decrease in FEV_1_ and PEF scores was not statistically significant. Use of *Nephrolepis exaltata* herbal mask significantly improved the pulmonary function. It is necessary to further study the potency of *Nephrolepis exaltata* herbal mask in sinonasal cellular immune system.

## Figures and Tables

**Table 1:** Characteristics of the subject (*N*=30).

Characteristics	Groups				*p^∗^*
	Control		Treatment		
	*N* (%)	Mean ± SD; median (min–max)	*N* (%)	Mean ± SD; median (min–max)	
Age		36.13 ± 9.49; 36 (22–53)		36.00 ± 7.00; 37 (23–44)	1.00

*Sex*					0.10
Male	11 (73.3%)		15 (100%)		
Female	4 (26.7%)		0 (0%)		

Length of work		15.4 ± 8.45; 19 (2–25)		15.6 ± 6.17; 17 (4–20)	0.68

*History of respiratory disease*					1.00
None	14 (93.3%)		14 (93.3%)		
Bronchitis	1 (6.7%)		1 (6.7%)		

*History of allergy*					0.22
None	12 (80.0%)		15 (100%)		
Food	2 (13.3%)		0 (0%)		
Drug	1 (6.7%)		0 (0%)		

*History of smoking*					0.06^∗^
No	12 (80.0%)		6 (40.0%)		
Yes	3 (20.0%)		9 (60.0%)		

∗Fisher's exacttest.

**Table 2:** IgA level.

IgA level	Groups	
	Control mean ± SD; Median (min–max)	Treatment mean ± SD; Median (min–max)
Before treatment (*Pre*)	3691.5 ± 325.01; 3755.4 (2965.0–4023.7)	3838.4 ± 172.3; 3882.4 (3522.7–4150.6)
After treatment (*Post*)	4656.9 ± 1345.66; 4948.7 (2097.3–6182.7)	4935.88 ± 608.01; 5209.1(3739.7–5627.4)
*p*	0.031∗	<0.001∗∗
Delta (after – before treatment)	965.3 ± 1411.37; 1180.9 (−1831.6–2800.1)	1097.5 ± 656.16; 1326.7 (−410.9–2011.8)

∗Wilcoxontest, ∗∗Paired *t*-test.

**Table 3:** Mean score of FVC before and after treatment.

Group	%FVC (Mean ± SD)			*p*∗	*p*∗∗
	Pre-test	Post-test	Δ FVC		
Control	93.3 ± 10.23	87.53 ± 8.38	−5.8 ± 9.09	0.033	0.006
Treatment	86.5 ± 10.74	90.0 ± 9.9	3.5 ± 6.14	0.038	

SD = Standard deviation, ∗ = Paired *t*-test, ∗∗ = Mann–Whitney test.

**Table 4:** Mean score of FEV_1_ before and after treatment.

Group	%FEV_1_ (Mean ± SD)			*p*∗	*p*∗∗
	Pre-test	Post-test	Δ FVC		
Control	100.9 ± 11.8	90.0 ± 7.8	−1.9 ± 10.3	0.507	0.009
Treatment	97.82 ± 11.87	102.5 ± 11.5	4.7 ± 5.5	0.004	

SD = Standard deviation, ∗ = Paired *t*-test, ∗∗ = Mann–Whitney test.

**Table 5:** Average score of PEF before and after treatment.

Group	%PEF (Mean ± SD)			*p*∗	*p*∗∗
	Pre-test	Post-test	Δ FVC		
Control	109.7 ± 20.54	106.2 ± 22.4	−3.4 ± 10.26	0.231	0.001
Treatment	114.1 ± 16.80	119.9 ± 15.2	5.8 ± 5.65	0.001		

SD = Standard deviation, ∗ = Paired *t*-test, ∗∗ = Mann–Whitney test.

## Data Availability

The word data used to support the findings of this study are included within the supplementary information file.

## Abbreviations

ALC: Alcoholic liver cirrhosis
ATP: Adenosine triphosphate
BBM: Bahan bakar mesin
BMI: Body mass index
BTX: Benzene, toluene, xylene
BTEX: Benzene, toluene, etilbenzene, xylene
CD: Cluster of differentiation
CpG: Cytosine-guanine dinucleotide
DIgA: Defisiensi imunoglobulin A
DNA: Deoxyribonucleic acid
ELISA: Enzyme-linked immunosorbent assay
FEV_1_: Forced expiratory volume in one second
FTIR: Fourier transform infrared
FVC: Forced vital capacity
HRP: Horseradish peroxidase
Ig: Immunoglobulin
IL: Interleukin
ILO: International labour organization
L: Litre
M: Meter
Mg: Milligrams
MALT: Mucosal-associated lymphoid tissue
MGUS: Monoclonal gammopathy of undetermined significance
MMFR: Maximum mid-expiratory flow rate
NHANES: National health and nutrition examination survey
NW: Nasal wash
OLD: Occupational lung disease
PAMP: Pathogen-associated molecular pattern
PEF: Peak expiratory flow
SC: Secretory component
SD: Standard deviation
SIg: Secretory immunoglobulin
SIgAD: Selective immunoglobulin A deficiency
TLR: Toll-like receptor
VOC: Volatile organic compound
VC: Vital capacity
WHO: World health organization.

## Supplementary Materials

Supplementary Materials (1) Descriptive data of the subjects, contain of age (ordinal), sex (nominal), length of work (ratio), History of Respiratory Disease (nominal), History of Allergy (nominal), History of smoking (nominal) (2) Mean Ig A level before and after treatment, control and tretment groups (numeric) (3) Mean score of FVC before and after treatment (pre and post test), control and tretment groups (numeric) (4) Mean score of FEV1 before and after treatment (pre and post test), control and tretment groups (numeric) (5) Average score of PEF before and after treatment (pre and post test), control and tretment groups (numeric) (6) INFORMED CONSENT (7) ETHICAL CLEARANCE.
