# Supplementary material for: Nephrolepis exaltata Herbal Mask Increases Nasal IgA Levels and Pulmonary Function in Textile Factory Workers
Source: Adv Prev Med. 2019 Dec 9;2019:5687135. doi: 10.1155/2019/5687135 (PMC6925762; doi:10.1155/2019/5687135)
Supplement: Supplementary Materials — (1) Descriptive data of the subjects, contain of age (ordinal), sex (nominal), length of work (ratio), History of Respiratory Disease (nominal), History of Allergy (nominal), History of smoking (nominal) (2) Mean Ig A level before and after treatment, control and tretment groups (numeric) (3) Mean score of FVC before and after treatment (pre and post test), control and tretment groups (numeric) (4) Mean score of FEV1 before and after treatment (pre and post test), control and tretment groups (numeric) (5) Average score of PEF before and after treatment (pre and post test), control and tretment groups (numeric) (6) INFORMED CONSENT (7) ETHICAL CLEARANCE. [file 5687135.f1.docx]

**SUPLEMENTARY MATERIAL
OUTPUT PENELITIAN MASKER HERBAL 2018**

**Frequencies**

**Frequency Table**

**Summarize**

**Crosstabs**

**Jenis Kelamin * Kelompok Masker**

**Tempat Kerja * Kelompok Masker**

**Paparan Lain * Kelompok Masker**

**Latihan Fisik Rutin * Kelompok Masker**

**Riwayat Gg Pernapasan * Kelompok Masker**

**Gejala Penyakit Pernapasan * Kelompok Masker**

**Riwayat Merokok * Kelompok Masker**

**Area Kerja * Kelompok Masker**

**Explore**

**Kelompok Masker**

**Usia**


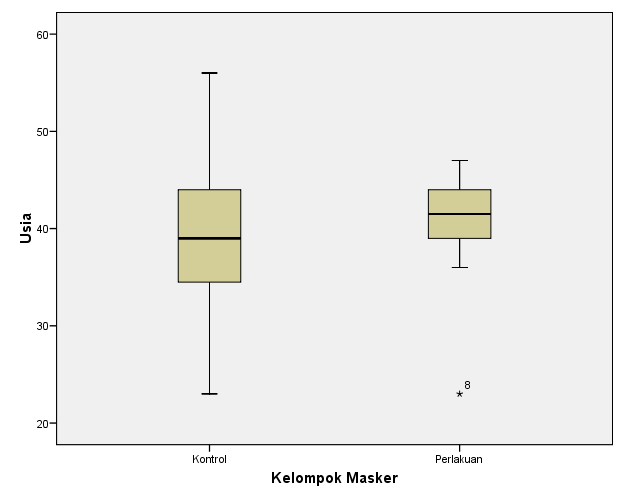


**Lama Bekerja (th)**


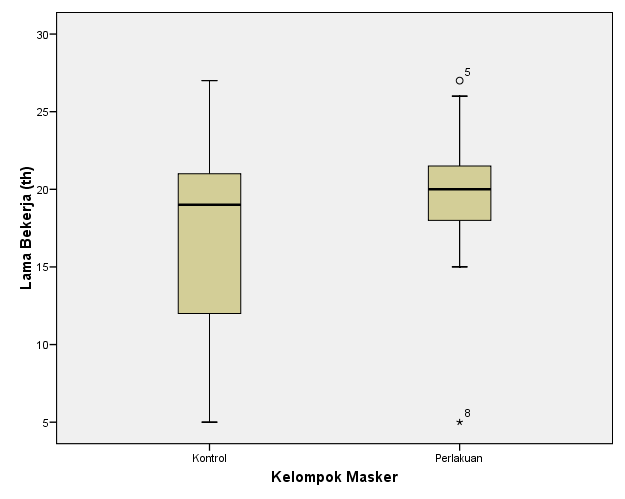


**Durasi Kerja (jam)**


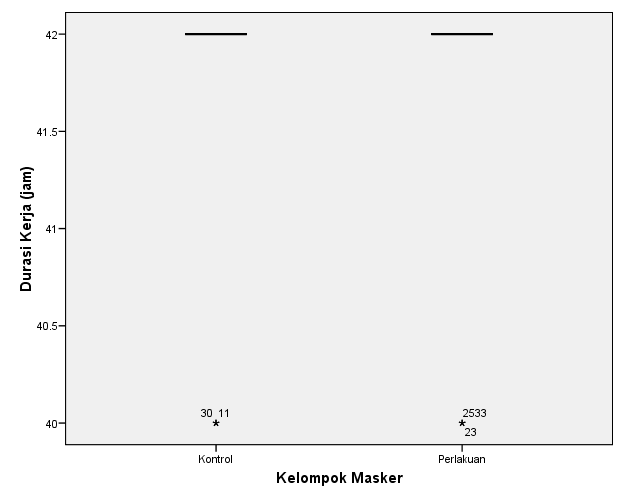


**NPar Tests**

**Mann-Whitney Test**

**Explore**

**Kelompok Masker**

**Reactive Oxygen Species Pre**


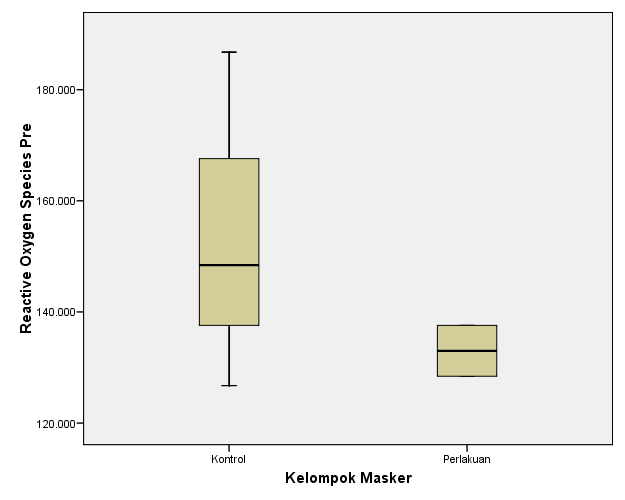


**Reactive Oxygen Species Post**


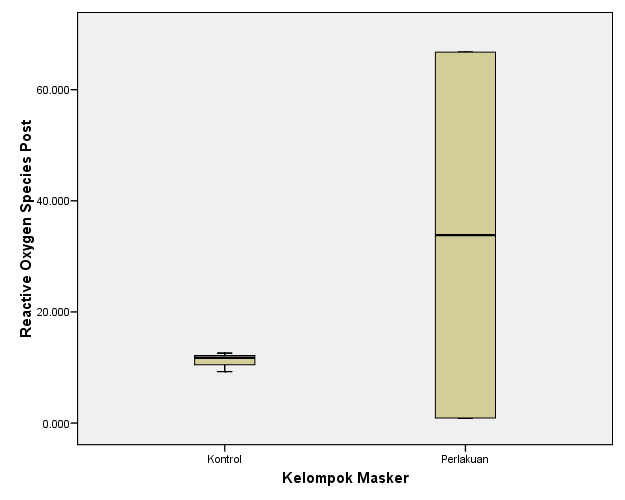


**Kontrol**

**T-Test**

**Perlakuan**

**T-Test**

**T-Test**

**T-Test**

**Explore**

**Kelompok Masker**

**Selisih Reactive Oxygen Species**


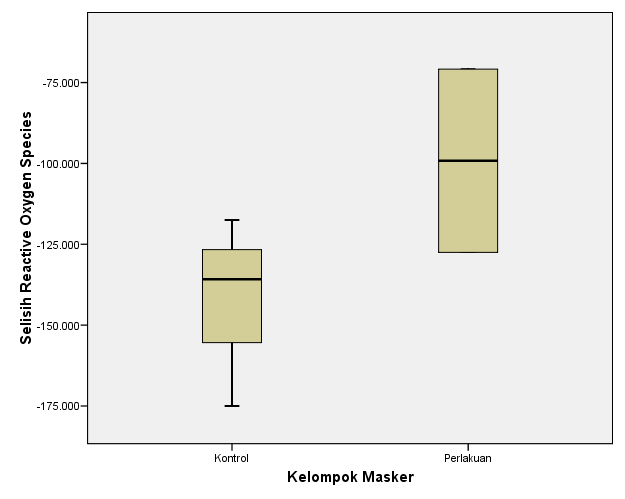


**T-Test**

**Explore SOD3**

**Kelompok Masker**

**Superoxide Dismutase 3 Pre**


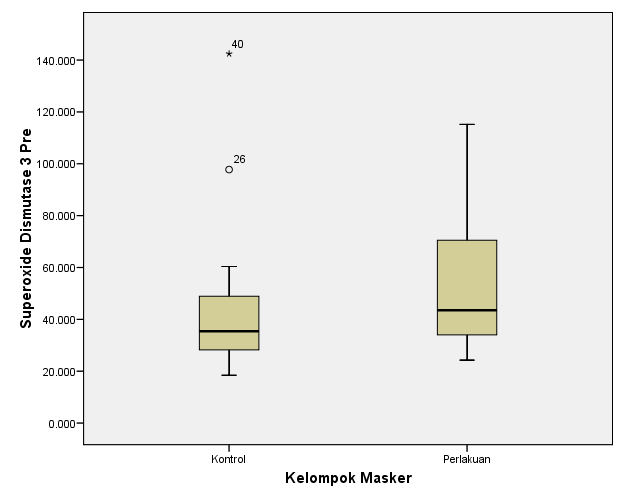


**Superoxide Dismutase 3 Post**


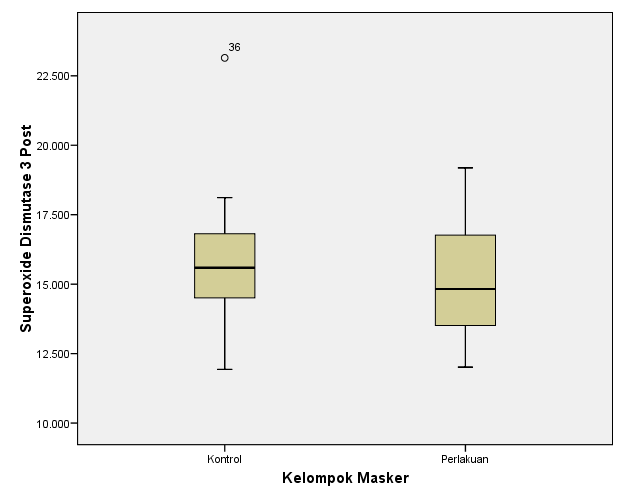


**Kontrol**

**NPar Tests**

**Wilcoxon Signed Ranks Test**

**Perlakuan**

**NPar Tests**

**Wilcoxon Signed Ranks Test**

**NPar Tests**

**Mann-Whitney Test**

**Explore**

**Kelompok Masker**

**Selisih Superoxide Dismutase 3**


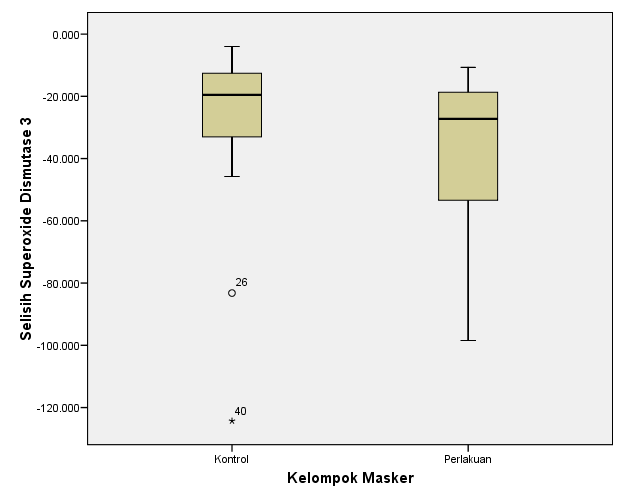


**NPar Tests**

**Mann-Whitney Test**

**Explore TNF-alfa**

**Kelompok Masker**

**Tumour Necrosys Alpha Pre**


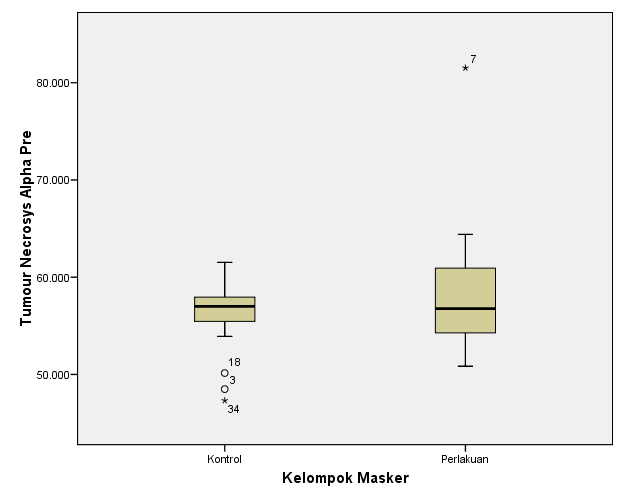


**Tumour Necrosys Alpha Post**


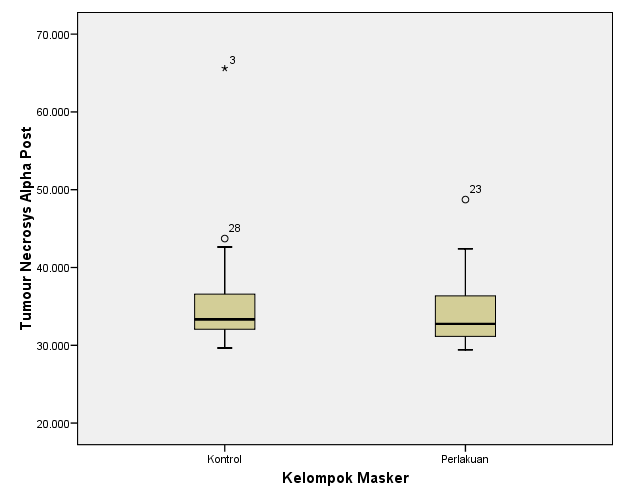


**Kontrol**

**NPar Tests**

**Wilcoxon Signed Ranks Test**

**Perlakuan**

**NPar Tests**

**Wilcoxon Signed Ranks Test**

**NPar Tests**

**Mann-Whitney Test**

**Explore**

**Kelompok Masker**

**Selisih Tumour Necrosys Alpha**


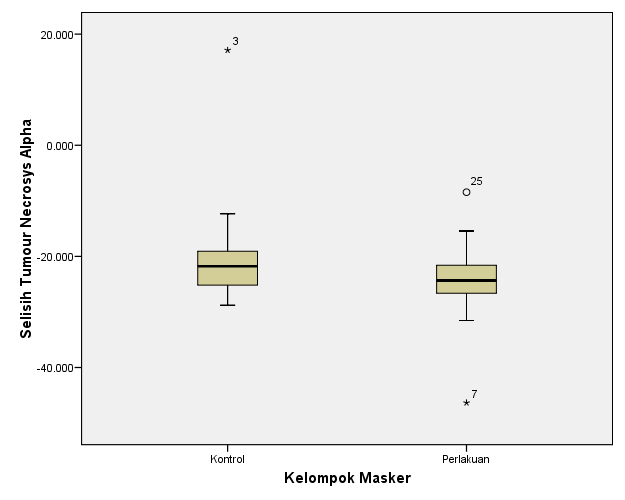


**NPar Tests**

**Mann-Whitney Test**

**Explore IL-6**

**Kelompok Masker**

**Interleukin 6 Pre**


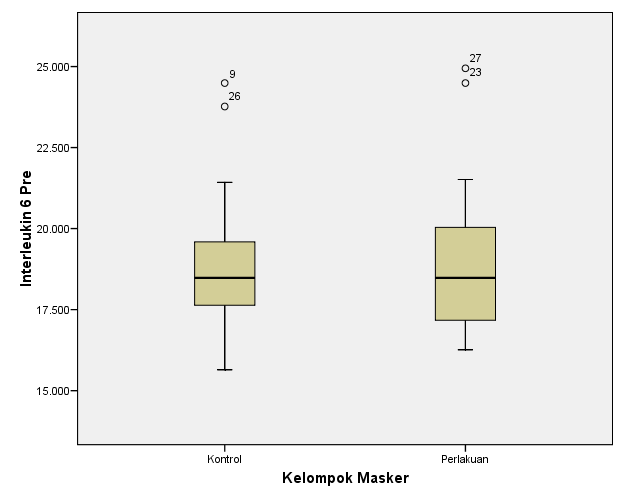


**Interleukin 6 Post**


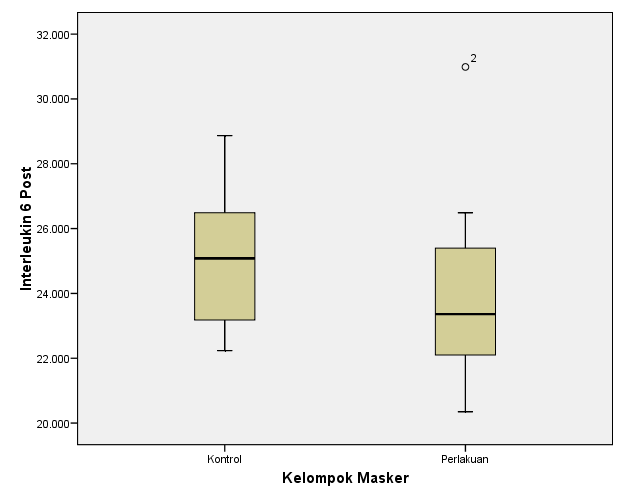


**Kontrol**

**T-Test**

**Perlakuan**

**NPar Tests**

**Wilcoxon Signed Ranks Test**

**NPar Tests**

**Mann-Whitney Test**

**T-Test**

**Explore**

**Kelompok Masker**

**Selisih Interleukin 6**


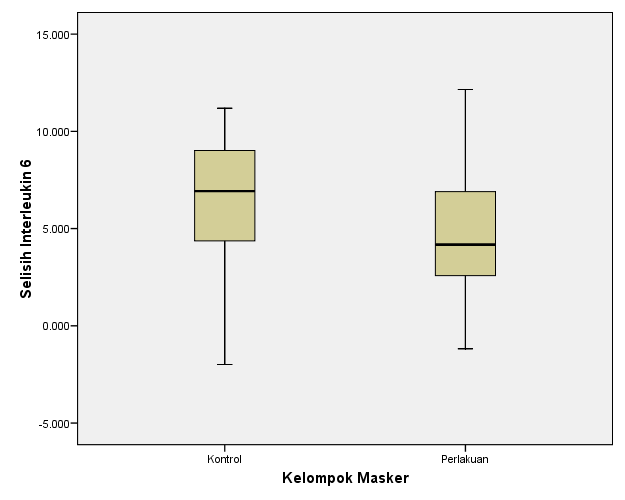


**T-Test**

**Explore IL-9**

**Kelompok Masker**

**Interleukin 9 Pre**


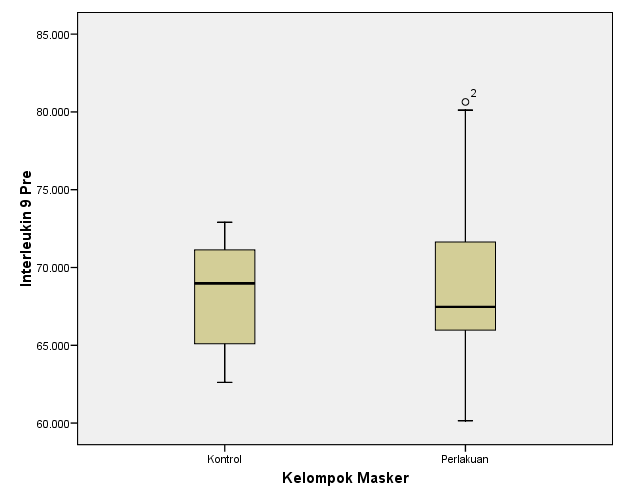


**Interleukin 9 Post**


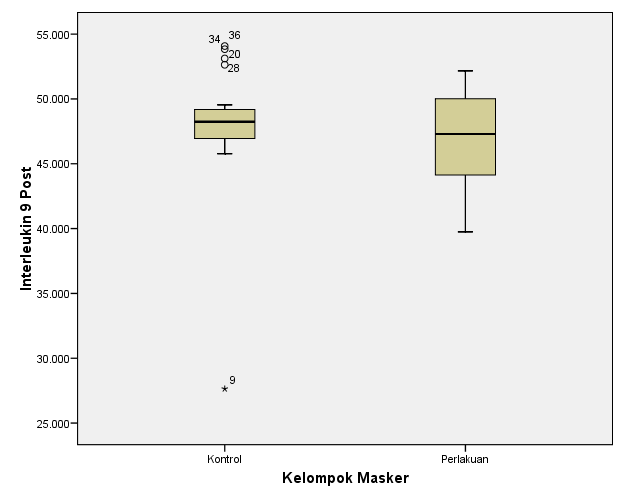


**Kontrol**

**NPar Tests**

**Wilcoxon Signed Ranks Test**

**Perlakuan**

**T-Test**

**T-Test**

**NPar Tests**

**Mann-Whitney Test**

**Explore**

**Kelompok Masker**

**Selisih Interleukin 9**


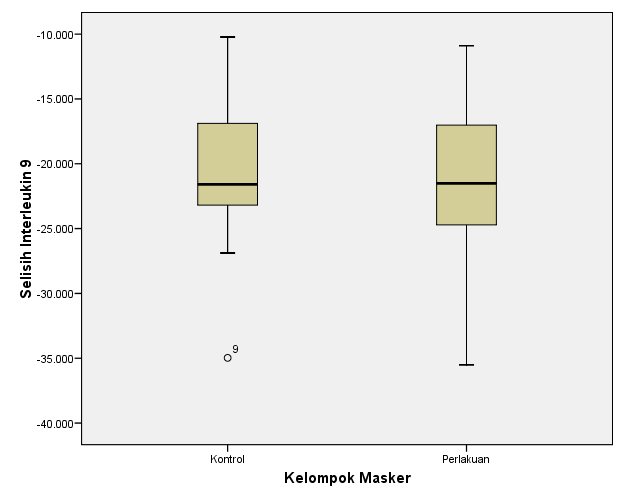


**T-Test**

**Explore**

**Kelompok Masker IL-13**

**Interleukin 13 Pre**


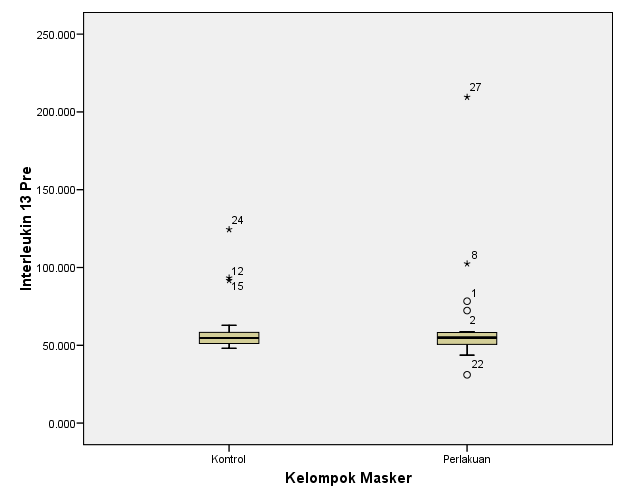


**Interleukin 13 Post**


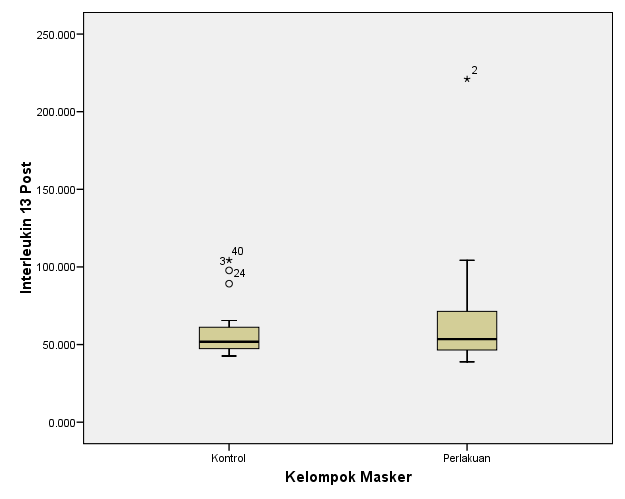


**Kontrol**

**NPar Tests**

**Wilcoxon Signed Ranks Test**

**Perlakuan**

**NPar Tests**

**Wilcoxon Signed Ranks Test**

**NPar Tests**

**Mann-Whitney Test**

**Explore**

**Kelompok Masker**

**Selisih Interleukin 13**


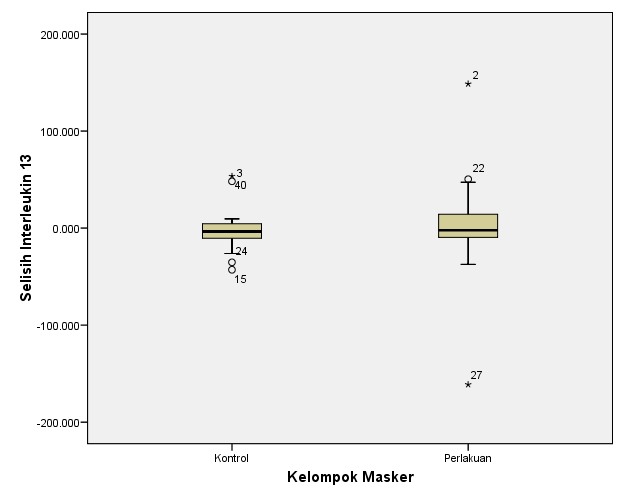


**NPar Tests**

**Mann-Whitney Test**

**Explore IgA**

**Kelompok Masker**

**Imunoglobulin A Pre**


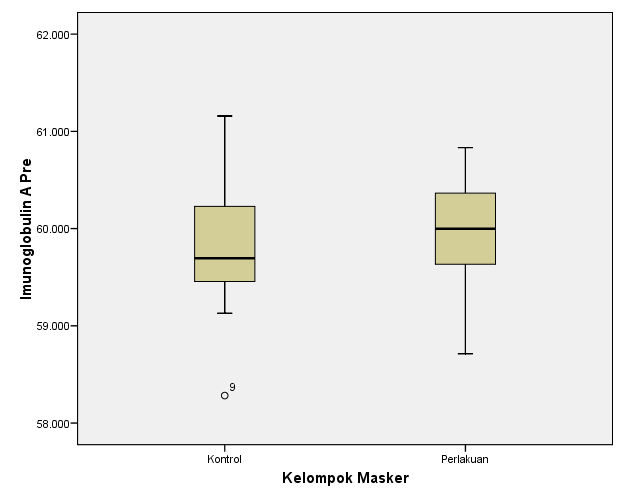


**Imunoglobulin A Post**


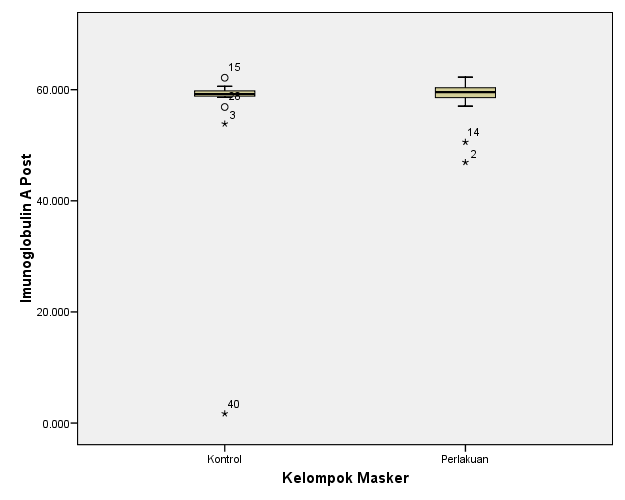


**Kontrol**

**NPar Tests**

**Wilcoxon Signed Ranks Test**

**Perlakuan**

**NPar Tests**

**Wilcoxon Signed Ranks Test**

**T-Test**

**NPar Tests**

**Mann-Whitney Test**

**Explore**

**Kelompok Masker**

**Selisih Imunoglobulin A**


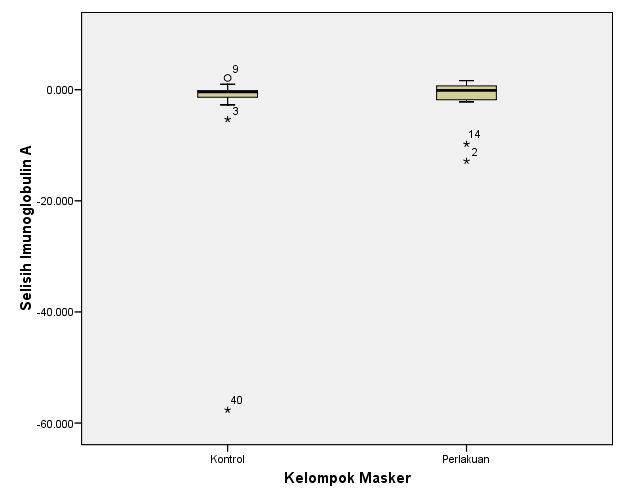


**NPar Tests**

**Mann-Whitney Test**

**Explore**

**Kelompok Masker**

**Transport Mukosiliar Hidung Pre (dtk)**


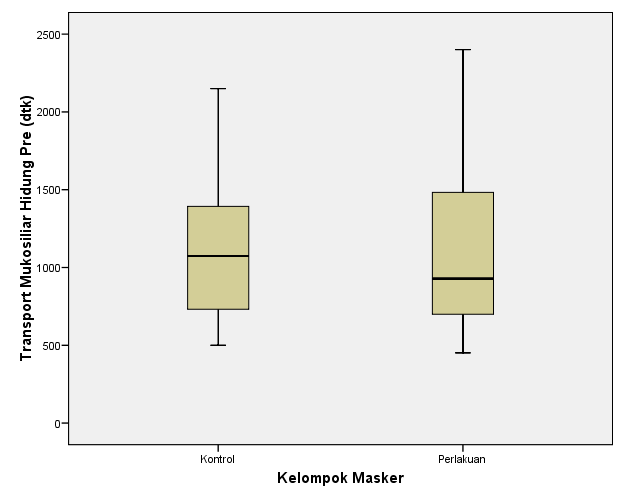


**Transport Mukosiliar Hidung Post (dtk)**


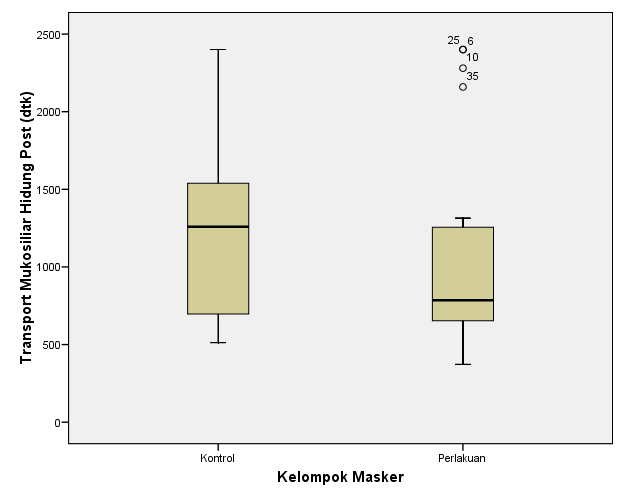


**Kontrol**

**T-Test**

**Perlakuan**

**NPar Tests**

**Wilcoxon Signed Ranks Test**

**NPar Tests**

**Mann-Whitney Test**

**Explore**

**Kelompok Masker**

**Selisih Transport Mukosiliar Hidung (dtk)**


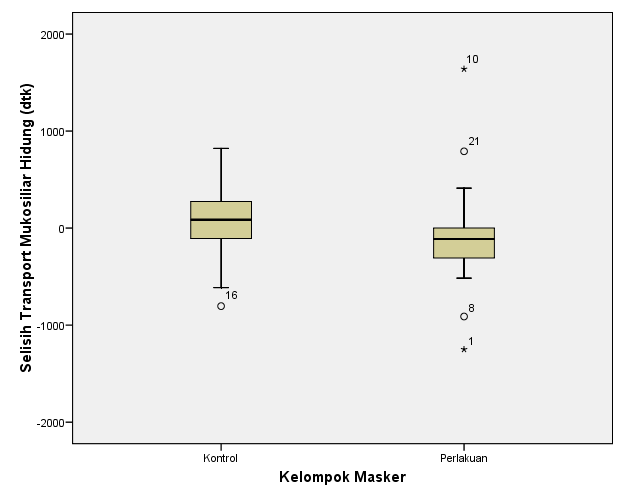


**NPar Tests**

**Mann-Whitney Test**

Tabel deskriptif data

| **Variabel** | **F** | **%** | **Mean ± SD** | **Median (min – max)** |
| --- | --- | --- | --- | --- |
| Kelompok Masker |  |  |  |  |
| Kontrol | 20 | 50 |  |  |
| Perlakuan | 20 | 50 |  |  |
| Jenis kelamin |  |  |  |  |
| Pria | 20 | 50 |  |  |
| Wanita | 20 | 50 |  |  |
| Usia |  |  | 39,70 ± 7,24 | 40 (23 – 56) |
| Lama bekerja (th) |  |  | 18,50 ± 6,22 | 20 (5 – 27) |
| Durasi kerja (jam) |  |  | 41,71 ± 0,72 | 42 (40 – 42) |
| Tempat kerja |  |  |  |  |
| Ruangan dengan ventilasi | 40 | 100 |  |  |
| Ruangan tanpa ventilasi | 0 | 0 |  |  |
| Paparan lain |  |  |  |  |
| Terpapar | 1 | 2,5 |  |  |
| Tidak terpapar | 39 | 97,5 |  |  |
| Latihan fisik rutin |  |  |  |  |
| Ya | 9 | 22,5 |  |  |
| Tidak | 31 | 77,5 |  |  |
| Riwayat gangguan pernapasan |  |  |  |  |
| Ada riwayat | 2 | 5,0 |  |  |
| Tidak ada riwayat | 38 | 95,0 |  |  |
| Gejala penyakit pernapasan |  |  |  |  |
| Ada gejala | 2 | 5,0 |  |  |
| Tidak ada gejala | 38 | 95,0 |  |  |
| Riwayat merokok |  |  |  |  |
| Merokok | 8 | 20,0 |  |  |
| Tidak merokok | 32 | 80,0 |  |  |
| Area kerja |  |  |  |  |
| Dyeing | 15 | 37,5 |  |  |
| Ball Warper | 8 | 20,0 |  |  |
| Sizing | 3 | 7,5 |  |  |
| Rebeamer | 10 | 25,0 |  |  |
| Rewinding | 4 | 10,0 |  |  |
| Reactive Oxygen Species Pre |  |  | 160,08 ± 18,38 | 159 (127 – 203) |
| Reactive Oxygen Species Post |  |  | 20,25 ± 26,40 | 11,75 (0,92 – 66,75) |
| Superoxide Dismutase 3 Pre |  |  | 49,40 ± 29,35 | 37,83 (18,44 – 142,42) |
| Superoxide Dismutase 3 Post |  |  | 15,43 ± 2,22 | 15,27 (11,93 – 23,14) |
| Tumour Necrosys Alpha Pre |  |  | 57,21 ± 5,43 | 57 (47,32 – 81,53) |
| Tumour Necrosys Alpha Pre |  |  | 35,12 ± 6,57 | 32,99 (29,41 – 65,6) |
| Interleukin 6 Pre |  |  | 18,90 ± 2,37 | 18,48 (15,65 – 24,94) |
| Interleukin 6 Post |  |  | 24,44 ± 2,39 | 23,99 (20,35 – 30,99) |
| Interleukin 9 Pre |  |  | 68,59 ± 4,48 | 68,6 (60,15 – 80,64) |
| Interleukin 9 Post |  |  | 47,43 ± 4,53 | 48 (27,65 – 54,08) |
| Interleukin 13 Pre |  |  | 62,79 ± 29,24 | 54,68 (31,05 – 209,52) |
| Interleukin 13 Post |  |  | 62,07 ± 30,83 | 52,32 (38,84 – 220,96) |
| Imunoglobulin A Pre |  |  | 59,88 ± 0,61 | 59,85 (58,28 – 61,16) |
| Imunoglobulin A Post |  |  | 57,45 ± 9,46 | 59,46 (1,71 – 62,26) |
| Transport Mukosiliar Hidung Pre |  |  | 1141,7 ± 561,4 | 978,5 (451 – 2400) |
| Transport Mukosiliar Hidung Post |  |  | 1131,6 ± 609,4 | 918 (372 – 2400) |

Tabel demografi data

| **Variabel** | **Kelompok Masker** | | **p** |
| --- | --- | --- | --- |
|  | **Kontrol** | **Perlakuan** |  |
| Jenis kelamin |  |  |  |
| Pria | 12 (60) | 8 (40) | 0,206^¥^ |
| Wanita | 8 (40) | 12 (60) |  |
| Usia | 39 (23 – 56) | 41,5 (23 – 47) | 0,254^‡^ |
| Lama bekerja (th) | 19 (5 – 27) | 20 (5 – 27) | 0,340^‡^ |
| Durasi kerja (jam) | 42 (40 – 42) | 42 (40 – 42) | 1,000^‡^ |
| Tempat kerja |  |  |  |
| Ruangan dengan ventilasi | 20 (100) | 20 (100) | – |
| Ruangan tanpa ventilasi | 0 (0) | 0 (0) |  |
| Paparan lain |  |  |  |
| Terpapar | 1 (5) | 0 (0) | 1,000^¥^ |
| Tidak terpapar | 19 (95) | 20 (100) |  |
| Latihan fisik rutin |  |  |  |
| Ya | 5 (25) | 4 (20) | 1,000^¥^ |
| Tidak | 15 (75) | 16 (80) |  |
| Riwayat gangguan pernapasan |  |  |  |
| Ada riwayat | 1 (5) | 1 (5) | 1,000^¥^ |
| Tidak ada riwayat | 19 (95) | 19 (95) |  |
| Gejala penyakit pernapasan |  |  |  |
| Ada gejala | 2 (10) | 0 (0) | 0,487^¥^ |
| Tidak ada gejala | 18 (90) | 20 (100) |  |
| Riwayat merokok |  |  |  |
| Merokok | 3 (15) | 5 (25) | 0,695^¥^ |
| Tidak merokok | 17 (85) | 15 (75) |  |
| Area kerja |  |  |  |
| Dyeing | 10 (50) | 5 (25) | 0,463^¥^ |
| Ball Warper | 4 (20) | 4 (20) |  |
| Sizing | 1 (5) | 2 (10) |  |
| Rebeamer | 3 (15) | 7 (35) |  |
| Rewinding | 2 (10) | 2 (10) |  |

Keterangan : ^¥^ Chi square; ^‡^ Mann whitney

Tabel diskriptif dan normalitas data Reactive Oxygen Species

| **Kelompok** | **Mean ± SD** | **Median (min – max)** | **p**^£^ |
| --- | --- | --- | --- |
| Kontrol |  |  |  |
| Pre | 153,97 ± 30,38 | 148,42 (126,75 – 186,75) | 0,696* |
| Post | 11,19 ± 1,73 | 11,75 (9,25 – 12,58) | 0,463* |
| Perlakuan |  |  |  |
| Pre | 133,00 ± 6,48 | 133 (128,42 – 137,58) | – |
| Post | 33,83 ± 46,55 | 33,83 (0,92 – 66,75) | – |

Keterangan : * Normal (p > 0,05); ^£^ Shapiro-wilk

Tabel perbedaan Reactive Oxygen Species pre dan post

| **Reactive Oxygen Species** | **Kelompok** | | **p** |
| --- | --- | --- | --- |
|  | **Kontrol** | **Perlakuan** |  |
| Pre | 153,97 ± 30,38 | 133,00 ± 6,48 | 0,427^§^ |
| Post | 11,19 ± 1,73 | 33,83 ± 46,55 | 0,425^§^ |
| p | 0,014^¶^* | 0,177^¶^ |  |

Keterangan : * Signifikan (p < 0,05); ^§^ Independent t; ^¶^ Paired t

Tabel diskriptif dan normalitas data selisih Reactive Oxygen Species

| **Kelompok** | **Mean ± SD** | **Median (min – max)** | **p**^£^ |
| --- | --- | --- | --- |
| Kontrol | -142,78 ± 29,37 | -135,83 (-175 – (-117,5)) | – |
| Perlakuan | -99,17 ± 40,07 | -99,17 (-127,5 – (-70,83)) | 0,606* |

Keterangan : * Normal (p > 0,05); ^£^ Shapiro-wilk

Tabel perbedaan selisih Reactive Oxygen Species kelompok kontrol dan perlakuan

| **Kelompok** | **Selisih Reactive Oxygen Species** | **p** |
| --- | --- | --- |
| Kontrol | -142,78 ± 29,37 | 0,247^§^ |
| Perlakuan | -99,17 ± 40,07 |  |

Keterangan : * Signifikan (p < 0,05); ^§^ Independent t

Tabel diskriptif dan normalitas data Superoxide Dismutase 3

| **Kelompok** | **Mean ± SD** | **Median (min – max)** | **p**^£^ |
| --- | --- | --- | --- |
| Kontrol |  |  |  |
| Pre | 44,28 ± 28,93 | 35,40 (18,44 – 142,42) | 0,000 |
| Post | 15,78 ± 2,34 | 15,59 (11,93 – 23,14) | 0,021 |
| Perlakuan |  |  |  |
| Pre | 54,51 ± 29,59 | 43,5 (24,26 – 115,17) | 0,005 |
| Post | 15,09 ± 2,10 | 14,83 (12,01 – 19,19) | 0,557* |

Keterangan : * Normal (p > 0,05); ^£^ Shapiro-wilk

Tabel perbedaan Superoxide Dismutase 3 pre dan post

| **Superoxide Dismutase 3** | **Kelompok** | | **p** |
| --- | --- | --- | --- |
|  | **Kontrol** | **Perlakuan** |  |
| Pre | 44,28 ± 28,93 | 54,51 ± 29,59 | 0,160^‡^ |
| Post | 15,78 ± 2,34 | 15,09 ± 2,10 | 0,387^‡^ |
| p | <0,001^†^* | <0,001^†^ |  |

Keterangan : * Signifikan (p < 0,05); ^‡^ Mann whitney; ^†^ Wilcoxon

Tabel diskriptif dan normalitas data selisih Superoxide Dismutase 3

| **Kelompok** | **Mean ± SD** | **Median (min – max)** | **p**^£^ |
| --- | --- | --- | --- |
| Kontrol | -28,51 ± 28,61 | -19,52 (-124,31 – (-3,98)) | 0,000 |
| Perlakuan | -39,43 ± 28,64 | -27,24 (-98,45 – (-10,67)) | 0,003 |

Keterangan : * Normal (p > 0,05); ^£^ Shapiro-wilk

Tabel perbedaan selisih Superoxide Dismutase 3 kelompok kontrol dan perlakuan

| **Kelompok** | **Selisih Superoxide Dismutase 3** | **p** |
| --- | --- | --- |
| Kontrol | -19,52 (-124,31 – (-3,98)) | 0,074^‡^ |
| Perlakuan | -27,24 (-98,45 – (-10,67)) |  |

Keterangan : * Signifikan (p < 0,05); ^‡^ Mann whitney

Tabel diskriptif dan normalitas data Tumour Necrosys Alpha

| **Kelompok** | **Mean ± SD** | **Median (min – max)** | **p**^£^ |
| --- | --- | --- | --- |
| Kontrol |  |  |  |
| Pre | 55,99 ± 3,59 | 57 (47,32 – 61,53) | 0,007 |
| Post | 35,89 ± 8,01 | 33,34 (29,64 – 65,6) | 0,000 |
| Perlakuan |  |  |  |
| Pre | 58,44 ± 6,67 | 56,77 (50,85 – 81,53) | 0,001 |
| Post | 34,35 ± 4,82 | 32,76 (29,41 – 48,73) | 0,002 |

Keterangan : * Normal (p > 0,05); ^£^ Shapiro-wilk

Tabel perbedaan Tumour Necrosys Alpha pre dan post

| **Tumour Necrosys Alpha** | **Kelompok** | | **p** |
| --- | --- | --- | --- |
|  | **Kontrol** | **Perlakuan** |  |
| Pre | 55,99 ± 3,59 | 58,44 ± 6,67 | 0,570^‡^ |
| Post | 35,89 ± 8,01 | 34,35 ± 4,82 | 0,570^‡^ |
| p | <0,001^†^* | <0,001^†^* |  |

Keterangan : * Signifikan (p < 0,05); ^‡^ Mann whitney; ^†^ Wilcoxon

Tabel diskriptif dan normalitas data selisih Tumour Necrosys Alpha

| **Kelompok** | **Mean ± SD** | **Median (min – max)** | **p**^£^ |
| --- | --- | --- | --- |
| Kontrol | -20,10 ± 9,76 | -21,77 (-28,79 – 17,11) | 0,000 |
| Perlakuan | -24,08 ± 7,18 | -24,34 (-46,34 – (-8,45)) | 0,009 |

Keterangan : * Normal (p > 0,05); ^£^ Shapiro-wilk

Tabel perbedaan selisih Tumour Necrosys Alpha kelompok kontrol dan perlakuan

| **Kelompok** | **Selisih Tumour Necrosys Alpha** | **p** |
| --- | --- | --- |
| Kontrol | -21,77 (-28,79 – 17,11) | 0,185^‡^ |
| Perlakuan | -24,34 (-46,34 – (-8,45)) |  |

Keterangan : * Signifikan (p < 0,05); ^‡^ Mann whitney

Tabel diskriptif dan normalitas data Interleukin 6

| **Kelompok** | **Mean ± SD** | **Median (min – max)** | **p**^£^ |
| --- | --- | --- | --- |
| Kontrol |  |  |  |
| Pre | 18,83 ± 2,36 | 18,48 (15,65 – 24,49) | 0,070* |
| Post | 25,08 ± 2,11 | 25,08 (22,23 – 28,87) | 0,204* |
| Perlakuan |  |  |  |
| Pre | 18,97 ± 2,44 | 18,48 (16,26 – 24,94) | 0,009 |
| Post | 23,80 ± 2,52 | 23,36 (20,35 – 30,99) | 0,061* |

Keterangan : * Normal (p > 0,05); ^£^ Shapiro-wilk

Tabel perbedaan Interleukin 6 pre dan post

| **Interleukin 6** | **Kelompok** | | **p** |
| --- | --- | --- | --- |
|  | **Kontrol** | **Perlakuan** |  |
| Pre | 18,83 ± 2,36 | 18,97 ± 2,44 | 0,935^‡^ |
| Post | 25,08 ± 2,11 | 23,80 ± 2,52 | 0,090^§^ |
| p | <0,001^¶^* | <0,001^†^* |  |

Keterangan : * Signifikan (p < 0,05) ; ^§^ Independent t; ^‡^ Mann whitney; ^¶^ Paired t; ^†^ Wilcoxon

Tabel diskriptif dan normalitas data selisih Interleukin 6

| **Kelompok** | **Mean ± SD** | **Median (min – max)** | **p**^£^ |
| --- | --- | --- | --- |
| Kontrol | 6,25 ± 3,41 | 6,93 (-1,99 – 11,19) | 0,408* |
| Perlakuan | 4,83 ± 3,23 | 4,18 (-1,18 – 12,15) | 0,970* |

Keterangan : * Normal (p > 0,05); ^£^ Shapiro-wilk

Tabel perbedaan selisih Interleukin 6 kelompok kontrol dan perlakuan

| **Kelompok** | **Selisih Interleukin 6** | **p** |
| --- | --- | --- |
| Kontrol | 6,25 ± 3,41 | 0,184^§^ |
| Perlakuan | 4,83 ± 3,23 |  |

Keterangan : * Signifikan (p < 0,05); ^§^ Independent t

Tabel diskriptif dan normalitas data Interleukin 9

| **Kelompok** | **Mean ± SD** | **Median (min – max)** | **p**^£^ |
| --- | --- | --- | --- |
| Kontrol |  |  |  |
| Pre | 68,36 ± 3,49 | 68,98 (62,62 – 72,91) | 0,090* |
| Post | 47,86 ± 5,40 | 48,24 (27,65 – 54,08) | 0,000 |
| Perlakuan |  |  |  |
| Pre | 68,82 ± 5,38 | 67,47 (60,15 – 80,64) | 0,262* |
| Post | 46,99 ± 3,54 | 47,30 (39,75 – 52,16) | 0,478* |

Keterangan : * Normal (p > 0,05); ^£^ Shapiro-wilk

Tabel perbedaan Interleukin 9 pre dan post

| **Interleukin 9** | **Kelompok** | | **p** |
| --- | --- | --- | --- |
|  | **Kontrol** | **Perlakuan** |  |
| Pre | 68,36 ± 3,49 | 68,82 ± 5,38 | 0,748^§^ |
| Post | 47,86 ± 5,40 | 46,99 ± 3,54 | 0,228^‡^ |
| p | <0,001^†^* | <0,001^¶^* |  |

Keterangan : * Signifikan (p < 0,05) ; ^§^ Independent t; ^‡^ Mann whitney; ^¶^ Paired t; ^†^ Wilcoxon

Tabel diskriptif dan normalitas data selisih Interleukin 9

| **Kelompok** | **Mean ± SD** | **Median (min – max)** | **p**^£^ |
| --- | --- | --- | --- |
| Kontrol | -20,49 ± 5,68 | -21,59 (-34,97 – (-10,23)) | 0,535* |
| Perlakuan | -21,83 ± 6,22 | -21,51 (-35,51 – (-10,9)) | 0,667* |

Keterangan : * Normal (p > 0,05); ^£^ Shapiro-wilk

Tabel perbedaan selisih Interleukin 9 kelompok kontrol dan perlakuan

| **Kelompok** | **Selisih Interleukin 9** | **p** |
| --- | --- | --- |
| Kontrol | -20,49 ± 5,68 | 0,481^§^ |
| Perlakuan | -21,83 ± 6,22 |  |

Keterangan : * Signifikan (p < 0,05); ^§^ Independent t

Tabel diskriptif dan normalitas data Interleukin 13

| **Kelompok** | **Mean ± SD** | **Median (min – max)** | **p**^£^ |
| --- | --- | --- | --- |
| Kontrol |  |  |  |
| Pre | 61,09 ± 19,43 | 54,68 (48,07 – 124,44) | 0,000 |
| Post | 58,89 ± 17,81 | 51,79 (42,6 – 104,29) | 0,000 |
| Perlakuan |  |  |  |
| Pre | 64,49 ± 37,04 | 54,89 (31,05 – 209,52) | 0,000 |
| Post | 65,25 ± 40,15 | 53,5 (38,84 – 220,96) | 0,000 |

Keterangan : * Normal (p > 0,05); ^£^ Shapiro-wilk

Tabel perbedaan Interleukin 13 pre dan post

| **Interleukin 13** | **Kelompok** | | **p** |
| --- | --- | --- | --- |
|  | **Kontrol** | **Perlakuan** |  |
| Pre | 61,09 ± 19,43 | 64,49 ± 37,04 | 0,968^‡^ |
| Post | 58,89 ± 17,81 | 65,25 ± 40,15 | 0,787^‡^ |
| p | 0,247^†^ | 0,911^†^ |  |

Keterangan : * Signifikan (p < 0,05) ; ^‡^ Mann whitney; ^†^ Wilcoxon

Tabel diskriptif dan normalitas data selisih Interleukin 13

| **Kelompok** | **Mean ± SD** | **Median (min – max)** | **p**^£^ |
| --- | --- | --- | --- |
| Kontrol | -2,20 ± 22,51 | -3,47 (-42,93 – 53,45) | 0,015 |
| Perlakuan | 0,75 ± 54,56 | -2,24 (-161,23 – 148,64) | 0,001 |

Keterangan : * Normal (p > 0,05); ^£^ Shapiro-wilk

Tabel perbedaan selisih Interleukin 13 kelompok kontrol dan perlakuan

| **Kelompok** | **Selisih Interleukin 13** | **p** |
| --- | --- | --- |
| Kontrol | -3,47 (-42,93 – 53,45) | 0,589^‡^ |
| Perlakuan | -2,24 (-161,23 – 148,64) |  |

Keterangan : * Signifikan (p < 0,05); ^‡^ Mann whitney

Tabel diskriptif dan normalitas data Imunoglobulin A

| **Kelompok** | **Mean ± SD** | **Median (min – max)** | **p**^£^ |
| --- | --- | --- | --- |
| Kontrol |  |  |  |
| Pre | 59,84 ± 0,67 | 59,7 (58,28 – 61,16) | 0,882* |
| Post | 56,27 ± 12,94 | 59,23 (1,71- 62,14) | 0,000 |
| Perlakuan |  |  |  |
| Pre | 59,92 ± 0,55 | 60 (58,71 – 60,83) | 0,717* |
| Post | 58,63 ± 3,62 | 59,55 (46,93 – 62,26) | 0,000 |

Keterangan : * Normal (p > 0,05); ^£^ Shapiro-wilk

Tabel perbedaan Imunoglobulin A pre dan post

| **Imunoglobulin A** | **Kelompok** | | **p** |
| --- | --- | --- | --- |
|  | **Kontrol** | **Perlakuan** |  |
| Pre | 59,84 ± 0,67 | 59,92 ± 0,55 | 0,690^§^ |
| Post | 56,27 ± 12,94 | 58,63 ± 3,62 | 0,285^‡^ |
| p | 0,009^†^* | 0,296^†^ |  |

Keterangan : * Signifikan (p < 0,05) ; ^§^ Independent t; ^‡^ Mann whitney; ^†^ Wilcoxon

Tabel diskriptif dan normalitas data selisih Imunoglobulin A

| **Kelompok** | **Mean ± SD** | **Median (min – max)** | **p**^£^ |
| --- | --- | --- | --- |
| Kontrol | -3,57 ± 12,81 | -0,45 (-57,64 – 2,11) | 0,000 |
| Perlakuan | -1,28 ± 3,66 | -0,12 – (-12,84 – 1,62) | 0,000 |

Keterangan : * Normal (p > 0,05); ^£^ Shapiro-wilk

Tabel perbedaan selisih Imunoglobulin A kelompok kontrol dan perlakuan

| **Kelompok** | **Selisih Imunoglobulin A** | **p** |
| --- | --- | --- |
| Kontrol | -0,45 (-57,64 – 2,11) | 0,417^‡^ |
| Perlakuan | -0,12 – (-12,84 – 1,62) |  |

Keterangan : * Signifikan (p < 0,05); ^‡^ Mann whitney

Tabel diskriptif dan normalitas data Transport Mukosiliar Hidung

| **Kelompok** | **Mean ± SD** | **Median (min – max)** | **p**^£^ |
| --- | --- | --- | --- |
| Kontrol |  |  |  |
| Pre | 1113,8 ± 479,4 | 1073,5 (500 – 2150) | 0,186* |
| Post | 1187,4 ± 545 | 1258,5 (512 – 2400) | 0,143* |
| Perlakuan |  |  |  |
| Pre | 1169,6 ± 644,6 | 928,5 (451 – 2400) | 0,004 |
| Post | 1075,8 ± 677,4 | 785 (372 – 2400) | 0,001 |

Keterangan : * Normal (p > 0,05); ^£^ Shapiro-wilk

Tabel perbedaan Transport Mukosiliar Hidung pre dan post

| **Transport Mukosiliar Hidung** | **Kelompok** | | **p** |
| --- | --- | --- | --- |
|  | **Kontrol** | **Perlakuan** |  |
| Pre | 1113,8 ± 479,4 | 1169,6 ± 644,6 | 1,000^‡^ |
| Post | 1187,4 ± 545 | 1075,8 ± 677,4 | 0,330^‡^ |
| p | 0,467^¶^ | 0,102^†^ |  |

Keterangan : * Signifikan (p < 0,05) ; ^‡^ Mann whitney; ^¶^ Paired t; ^†^ Wilcoxon

Tabel diskriptif dan normalitas data selisih Transport Mukosiliar Hidung

| **Kelompok** | **Mean ± SD** | **Median (min – max)** | **p**^£^ |
| --- | --- | --- | --- |
| Kontrol | 73,65 ± 443,78 | 86,5 (-804 – 821) | 0,442* |
| Perlakuan | -93,85 ± 588,63 | -112 (-1250 – 1640) | 0,014 |

Keterangan : * Normal (p > 0,05); ^£^ Shapiro-wilk

Tabel perbedaan selisih Transport Mukosiliar Hidung kelompok kontrol dan perlakuan

| **Kelompok** | **Selisih Transport Mukosiliar Hidung** | **p** |
| --- | --- | --- |
| Kontrol | 86,5 (-804 – 821) | 0,048^‡*^ |
| Perlakuan | -112 (-1250 – 1640) |  |

Keterangan : * Signifikan (p < 0,05); ^‡^ Mann whitney
